# Association between Psychosocial Stress and Fecal Microbiota in Pregnant Women

**DOI:** 10.1038/s41598-019-40434-8

**Published:** 2019-03-14

**Authors:** C. Hechler, K. Borewicz, R. Beijers, E. Saccenti, M. Riksen-Walraven, H. Smidt, C. de Weerth

**Affiliations:** 1 Behavioral Science Institute, Radboud University Nijmegen, Montessorilaan 3, 6525 HR Nijmegen, The Netherlands; 2 Laboratory of Microbiology, Wageningen University & Research, Stippeneng 4, 6708 WE Wageningen, The Netherlands; 3 Laboratory of Systems and Synthetic Biology, Wageningen University & Research, Stippeneng 4, 6708 WE Wageningen, The Netherlands; 4 Department of Cognitive Neuroscience, Donders Institute for Brain, Cognition and Behaviour, Radboud University Medical Center, Kapittelweg 29, 6525 EN Nijmegen, The Netherlands

## Abstract

Maternal prenatal psychosocial stress is associated with altered child emotional and behavioral development. One potential underlying mechanism is that prenatal psychosocial stress affects child outcomes via the mother’s, and in turn the child’s, intestinal microbiota. This study investigates the first step of this mechanism: the relation between psychosocial stress and fecal microbiota in pregnant mothers. Mothers (N = 70) provided a late pregnancy stool sample and filled in questionnaires on general and pregnancy-specific stress and anxiety. Bacterial DNA was extracted and analysed by Illumina HiSeq sequencing of PCR-amplified 16 S ribosomal RNA gene fragments. Associations between maternal general anxiety and microbial composition were found. No associations between the other measured psychosocial stress variables and the relative abundance of microbial groups were detected. This study shows associations between maternal pregnancy general anxiety and microbial composition, providing first evidence of a mechanism through which psychological symptoms in pregnancy may affect the offspring.

## Introduction

Accumulating evidence indicates that maternal psychosocial stress during pregnancy may affect child emotional, behavioral and cognitive development, as well as physical health^1–3^. Psychosocial stress can be defined as demanding conditions, including stressful life events and antenatal depression, that exceed behavioral resources^4^. During pregnancy it includes maternal general and pregnancy-specific stress and anxiety^5^. Maternal psychosocial stress during pregnancy has been related to worse birth outcomes, including lower birth weight and shorter gestational age, as well as to compromised offspring cognitive and neurological development, difficult temperament, and increased risk of psychiatric disorders in numerous epidemiological and case-control studies^6^. Nevertheless, the mechanisms underlying the relations between prenatal psychosocial stress and child outcomes are only partly understood^5^.

The most investigated mechanism to explain the relations between maternal prenatal psychosocial stress and child outcomes is increased hypothalamic–pituitary–adrenal axis (HPA axis) activation, resulting in increased cortisol concentrations that could harm the developing fetus^7^. Other mechanisms that received attention are increased catecholamines, impaired placental functioning, compromised maternal immunity, including increased inflammation, and altered maternal health behaviors including eating, sleep, and exercise^5^. One less studied underlying mechanism is that prenatal psychosocial stress affects the child via the mother’s, and in turn the infant’s intestinal microbiota^8^. The intestinal bacteria have a central position in human health and disease and are suggested to also play a role in the development of emotion regulation, behavior, and higher cognitive functions^8^. To our knowledge, this study is the first to investigate an essential part of this potential mechanism, namely the relation between psychosocial stress and fecal microbiota in pregnant mothers.

The microorganisms important for the colonization of the children’s gut originate mainly from the mother. While major colonization of the neonate’s intestines commences at delivery^9,10^, there are indications that the intrauterine environment may not be sterile, and that there may already be transmission of bacteria from mother to fetus through the placenta^11^. If the maternal microbiota is unbalanced, e.g. as a possible result of psychosocial stress, infant intestinal colonization might be altered, with possible consequences for child mental and physical development^12,13^.

Results from rodent and primate models support the link between prenatal psychosocial stress and offspring intestinal microbiota^14–16^. Additionally, one human study found that infant intestinal microbiota from mothers with high prenatal psychosocial stress was characterized by more Proteobacteria, and lower levels of Actinobacteria and lactobacilli^17^.

Indications that psychosocial stress might be related to fecal microbiota during pregnancy come from a mouse study showing that stress during pregnancy was associated with changes in the gut microbiota^18^, and from a study in non-pregnant mice, where exposure to a social stressor led to decreased relative abundance of bacteria in the genus *Bacteroides* and increased relative abundance of bacteria in the genus *Clostridium*^19^. In non-pregnant humans, physiological and psychological stress negatively affects the intestinal microbiota^20^, and is related to gastrointestinal illnesses such as irritable bowel syndrome^21^. Additionally, there is evidence that maternal psychosocial stress might alter maternal vaginal microbiota in humans^16^; the same may be true for intestinal microbiota. Finally, in a study on healthy non-pregnant female students, the concentration of beneficial lactic acid bacteria was lower during a stressful week (first week of exams) as compared to a low-stress week (beginning of semester)^20^.

The current study examined associations between maternal psychosocial stress and intestinal microbiota composition in late pregnancy. Based on the findings by^17^, we hypothesized that mothers with high psychosocial stress would have phylum-level microbial compositions characterized by more Proteobacteria, and less Actinobacteria, compared to mothers with low reported psychosocial stress. We additionally explored potential differences at genus-level, where we hypothesized to find lower levels of lactobacilli in mothers with high psychosocial stress.

## Results

### Descriptive Statistics

**Figure 1:**
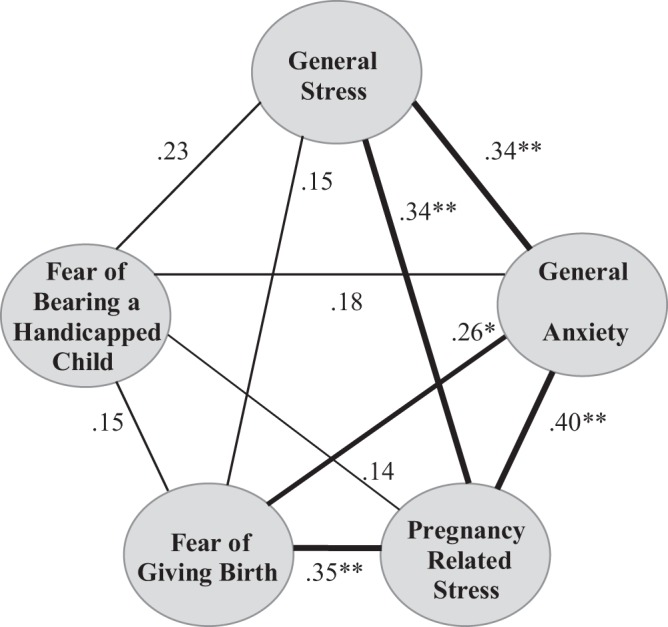
Correlations between the psychosocial stress variables (**p < 0.001, * < 0.05). Note that Fear of Bearing a Handicapped Child and Fear of Giving Birth are considered pregnancy related anxieties.

Descriptive statistics for the study variables can be found in Table 1. Figure 1 shows the Pearson correlations between the psychosocial stress variables. General stress was positively correlated with general anxiety (*r* = 0.34, *p* = 0.005) and pregnancy-related stress (*r* = 0.34, *p* = 0.005). Furthermore, general anxiety was positively related with pregnancy-related stress (*r* = 0.40, *p* = 0.001) and fear of giving birth (*r* = 0.26, *p* = 0.027), and pregnancy-related stress was positively related with fear of giving birth (*r* = 0.35, *p* = 0.003). The strength of these correlations was weak, indicating that despite these associations, the variables generally tap into different aspects of maternal psychosocial stress. Correlations between maternal characteristics and psychosocial stress can be found in Table 2.

**Table 1:** Descriptive Statistics for the Study Variables.

	*M (SD)*	Range	N
Age (in years)	31.61(*3*.*66*)	25.36–40.82	70
**Educational background**			70
College			21 (29%)
University			39 (54%)
**Parity**			
First child	n = 59		
Second child	n = 11		
**Civil state**			
Married	n = 36		
Cohabiting, not married	n = 34		
**Gestational age at collection** (in weeks)	33.60(*2*.*38*)	28.00–38.86	62^a^
**Prenatal psychosocial stress**			
General stress (EPL)	2.22(*0*.*42*)	1.36–3.14	68^a^
General anxiety (STAI)	29.97(*6*.*06*)	20–45	70
Pregnancy-related stress (PES)	0.29(*0*.*21*)	0–0.87	70
Fear of giving birth (PRAQ-R)	5.76(*2*.*11*)	3–11	70
Fear of bearing a handicapped child (PRAQ-R)	8.91(*2*.*94*)	4–20	70

^a^Some participants did not fill in all questionnaires, hence N < 70.

**Table 2:** Correlations Between Maternal Characteristics and Psychosocial Stress.

	General Stress	General Anxiety	Pregnancy-related stress	Fear of giving birth	Fear of bearing a handicapped child
Age	−0.11	−0.12	−0.10	−0.27*	−0.06
Education	−0.15	−0.04	0.10	−0.04	0.06
Parity	−0.08	0.09	−0.12	−0.33**	−0.06
Civil state	0.08	0.01	0.02	−0.03	−0.12
Gestational age at collection	0.17	0.16	0.01	0.05	−0.03

Note. *p < 0.05, **p < 0.001.

Fecal samples from 70 mothers were analyzed for microbial composition using Illumina HiSeq sequencing of barcoded 16 S rRNA gene amplicons. The total number of resulting sequencing reads was 10,201,505 and ranged from 6,447 to 632,101 reads per sample with an average number of reads per sample of 139,747 (Std. Deviation = 130,752, Std. Error = 15,303). A total of 113 genus level taxa were identified, of which 76 were present in more than 95% of all samples. The average relative abundance of these taxa is summarized in Table 3.

**Table 3:** Genus Level Taxa Detected in at Least 95% of the Samples from the 70 Mothers.

Genus Taxon	Average RA (%)	SE	Prevalence	Genus Taxon	Average RA (%)	SE	Prevalence
*Blautia*	13.38	1.6E-02	100.0	*Methanosphaera*	0.063	7.6E-05	8.6
*Faecalibacterium*	7.12	8.5E-03	100.0	*Bilophila*	0.056	6.7E-05	25.7
*Ruminococcus*	6.61	7.9E-03	95.7	*Peptococcus*	0.055	6.5E-05	18.6
*Bifidobacterium*	4.86	5.8E-03	100.0	*Slackia*	0.053	6.3E-05	11.4
*Pseudobutyrivibrio*	4.08	4.9E-03	97.1	*Megasphaera*	0.040	4.8E-05	10.0
*Prevotella*	4.32	5.2E-03	48.6	RC9_gut_group	0.022	2.6E-05	7.1
*Subdoligranulum*	4.03	4.8E-03	97.1	*Gordonibacter*	0.034	4.1E-05	21.4
*Bacteroides*	3.59	4.3E-03	95.7	*Thalassospira*	0.032	3.9E-05	14.3
*Coprococcus*	2.81	3.4E-03	98.6	*Acidaminococcus*	0.027	3.3E-05	8.6
*Anaerostipes*	2.58	3.1E-03	98.6	*Veillonella*	0.023	2.8E-05	10.0
*Dorea*	1.85	2.2E-03	100.0	*Enterorhabdus*	0.020	2.3E-05	10.0
*Streptococcus*	1.48	1.8E-03	91.4	*Halomonas*	0.021	2.5E-05	12.9
*Roseburia*	1.39	1.7E-03	97.1	*Haemophilus*	0.017	2.0E-05	7.1
*Methanobrevibacter*	1.27	1.5E-03	37.1	*Actinomyces*	0.011	1.3E-05	8.6
*Akkermansia*	1.15	1.4E-03	60.0	*Butyricimonas*	0.014	1.7E-05	7.1
*Clostridium*	1.11	1.3E-03	78.6	*Aeribacillus*	0.008	9.3E-06	7.1
*Phascolarctobacterium*	1.11	1.3E-03	55.7	f_S24-7_g_g	0.786	9.4E-04	38.6
*Dialister*	1.08	1.3E-03	52.9	f_Ruminococcaceae_*Incertae_Sedis*	1.202	1.4E-03	100.0
*Alistipes*	0.72	8.7E-04	77.1	f_Ruminococcaceae_g_g	6.275	7.5E-03	100.0
*Lachnospira*	0.53	6.3E-04	75.7	f_Prevotellaceae_g_g	0.178	2.1E-04	11.4
*Parabacteroides*	0.46	5.5E-04	77.1	f_Peptostreptococcaceae_*Incertae_Sedis*	2.285	2.7E-03	94.3
*Barnesiella*	0.46	5.4E-04	65.7	f_Peptococcacea_g_g	0.018	2.1E-05	11.4
*Turicibacter*	0.37	4.4E-04	42.9	f_Lachnospiracaea_*Incertae_Sedis*	8.887	1.1E-02	100.0
*Catenibacterium*	0.35	4.2E-04	10.0	f_Lachnospiraceae_g_g_	3.980	4.8E-03	100.0
*Collinsella*	0.23	2.8E-04	18.6	f_Family_XIII_*Incertia-Sedis*	0.164	2.0E-04	44.3
*Marvinbryantia*	0.22	2.6E-04	71.4	f_Erysipelotrichaceae_g_g	2.205	2.6E-03	98.6
*Paraprevotella*	0.17	2.0E-04	38.6	f_Erysipelotrichaceae_*Incertae_Sedis*	1.072	1.3E-03	62.9
*Lactococcus*	0.17	2.0E-04	17.1	f_Enterobacteriaceae_g_g	0.161	1.9E-04	8.6
*Butyrivibrio*	0.17	2.0E-04	12.9	f_Coriobacteriaceae_g_g	0.633	7.6E-04	87.1
*Adlercreutzia*	0.16	1.9E-04	45.7	f_Christensenellaceae_g_g	1.854	2.2E-03	95.7
*Sutterella*	0.15	1.8E-04	37.1	C_4C0d-2_g_g	0.245	2.9E-04	30.0
*Desulfovibrio*	0.14	1.7E-04	31.4	o_vadinHA64_g_g	0.043	5.2E-05	10.0
*Escherichia-Shigella*	0.13	1.6E-04	31.4	o_RF9_g_g	0.489	5.8E-04	55.7
*Odoribacter*	0.09	1.1E-04	41.4	o_Coriobacteriales_g_g	0.028	3.3E-05	7.1
*Lactobacillus*	0.09	1.1E-04	17.1	o_Clostridiales_f_uncultured_g_g	0.018	2.2E-05	8.6
*Eggerthella*	0.03	3.1E-05	10.0	o_Clostridiales_g_g	0.023	2.7E-05	12.9
*Anaerotruncus*	0.08	9.4E-05	28.6	o_Bacteroidales_g_g	0.039	4.7E-05	11.4
*Parasutterella*	0.05	6.4E-05	17.1	k_NA	0.017	2.0E-05	7.1
Total genus level taxa (n = 113)				Other^a^ (n = 37)	0.321	5.4E-04	53.0

Note. When a taxonomic assignment could not be made at genus level, the lowest classifiable taxonomy assignment (family-f, class-c, order-o, kingdom-k) was used, and the unidentified genus with that level was denoted as “g_g”.

RA = Relative Abundance, SE = Standard Error, ^a^Genera found in less than 95% of the samples.

### Associations between maternal prenatal psychosocial stress and microbiota

Partial least squares (PLS) modeling was performed to predict the psychosocial stress variables from microbiota relative abundance profiles at phylum level. No significant associations were found between any of the maternal psychosocial stress variables and the microbiota at phylum level (Table 4).

**Table 4:** Statistics for the PLS Regression at Phylum Level.

Response (y)	*R* ^2^	*p*
General stress (EPL)	0.05	1
General anxiety (STAI)	0.13	0.95
Pregnancy-related stress (PES)	0.04	1
Fear of giving birth (PRAQ-R)	0.06	1
Fear of bearing a handicapped child (PRAQ-R)	0.29	0.40

Note. Analyses were corrected for multiple testing with Bonferroni correction.

PLS modeling was repeated at genus level. These statistical analyses showed that general anxiety (STAI) was significantly associated with microbial relative abundance profiles in mothers (*R*^2^ = 0.71, *p* = 0.04). There were no associations between microbiota relative abundance profiles at genus level and the other psychosocial stress variables (Table 5).

**Table 5:** Statistics for the PLS Regression at Genus Level.

Response (y)	*R* ^2^	*p*
General stress (EPL)	0.51	1
General anxiety (STAI)	0.71	0.04
Pregnancy-related stress (PES)	0.48	1
Fear of giving birth (PRAQ-R)	0.53	1
Fear of bearing a handicapped child (PRAQ-R)	0.65	0.06

Note. Analyses were corrected for multiple testing with Bonferroni correction.

The Significance Multivariate Correlation criterion was used to select the taxa contributing to the predictive model of general anxiety. *Parasutterella* was found to significantly contribute to the model at the 0.01 significance level. Ten genus level groups were significant at the 0.05 level, and the average relative abundance of these microbial taxa was calculated for the mothers with high (above the median of 30) and low (below 30) general anxiety (Table 6). The fecal microbiota of mothers with lower prenatal anxiety was characterized by higher relative abundance of *Oscillospira*, *Eubacterium*, and *Megamonas*. The fecal microbiota of mothers with higher prenatal anxiety was characterized by higher relative abundance of *Oxalobacter*, *Rothia*, *Acetitomaculum*, *Acidaminococcus*, and *Staphylococcus*, and unclassified genus-level taxa within the families *Peptococcaceae* and *Peptostreptococcaceae*.

**Table 6:** Microbial Taxa Significantly Associated at p < 0.05-level with High and Low General Anxiety Groups.

Phylum	Class	Order	Family	Genus	Low general anxiety			High general anxiety		
					*Mean RA*	*SE*	*Prevalence*	*Mean RA*	*SE*	*Prevalence*
Firmicutes	Clostridia	Clostridiales	Lachnospiraceae	*Acetitomaculum*	0.00007	0.0004	1	0.00009	0.0004	2
Firmicutes	Clostridia	Clostridiales	Veillonellaceae	*Acidaminococcus*	0.00004	0.0002	1	0.00049	0.0022	4
Firmicutes	Clostridia	Clostridiales	Eubacteriaceae	*Eubacterium*	0.00007	0.0004	1	n.d.		0
Firmicutes	Clostridia	Clostridiales	Peptococcaceae	unidentified	0.00003	0.0020	1	0.00030	0.0007	6
Firmicutes	Clostridia	Clostridiales	Peptostreptococcaceae	*Incertae_Sedis*	0.01919	0.0172	32	0.02560	0.0361	34
Firmicutes	Clostridia	Clostridiales	Veillonellaceae	*Megamonas*	0.00046	0.0022	2	n.d.		0
Firmicutes	Clostridia	Clostridiales	Ruminococcaceae	*Oscillospira*	0.00004	0.0002	1	n.d.		0
Proteobacteria	Betaproteobacteria	Burkholderiales	Oxalobacteraceae	*Oxalobacter*	n.d.		0	0.00007	0.0004	1
Actinobacteria	Actinobacteria	Micrococcales	Micrococcaceae	*Rothia*	n.d.		0	0.00004	0.0003	1
Firmicutes	Bacilli	Bacillales	Staphylococcaceae	*Staphylococcus*	0.00003	0.0020	1	0.00004	0.0003	1
Proteobacteria	Betaproteobacteria	Burkholderiales	Sutterellaceae	*Parasutterella* ^a^	0.00072	0.0022	5	0.00041	0.0010	7

Note. RA = Relative Abundance, SE = Standard Error, n.d. = not detected, Prevalence = number of samples where genus was found, ^a^Was significant at *p* < 0.01 level.

Two tailed, unpaired T- tests showed no differences in microbial richness (i.e., how many genera there are in each mother) and diversity (i.e., number and relative abundance distribution of genera) as respectively estimated with the Chao1 richness and Shannon diversity scores, between mothers in the high and low general anxiety groups (*p* = 0.92 and *p* = 0.88, respectively). PCoA based on weighted and unweighted unifrac distances as well as PCA and RDA analyses based on relative abundance data were used to see whether the overall microbiota profiles of mothers with low and high general anxiety were similar or not. None of these analyses showed separation of the data in relation to low/high general anxiety (data not shown). Thus, the microbiota of mothers with low and high general anxiety showed no specific patterns overall.

## Discussion

The current study investigated an essential step of the proposed mechanism behind the links between maternal pregnancy psychosocial stress and child outcomes: the relation between psychosocial stress and fecal microbiota in pregnant mothers. Based on previous findings^17^, mothers with high self-reported psychosocial stress during pregnancy were hypothesized to have fecal microbial profiles characterized by a higher relative abundance of Proteobacteria and lower relative abundance of Actinobacteria and lactobacilli, as compared to mothers with low reported psychosocial stress. This hypothesis was not confirmed, as in our study sample there were no differences in microbial profiles at phylum level based on psychosocial stress. Our additional exploratory analyses, however, revealed that several microbial taxa at genus level significantly contributed to a PLS model for the prediction of general anxiety during pregnancy. These taxa, however, did not include the genus *Lactobacillus*, which had previously been found to be reduced in relative abundance in mothers with high prenatal psychosocial stress^17^. Finally, we did not find significant associations between general stress and pregnancy-specific stress and anxiety and maternal fecal microbiota composition at genus level.

We assumed that prenatal psychosocial stress might affect the child’s development via the mother’s, and in turn the infant’s intestinal microbiota^5^, as the largest bacterial colonization of the infants’ intestines occurs after transfer of maternal-origin bacteria during vaginal delivery^9,10^. Furthermore, infants’ intestinal bacteria have been shown to differ based on the level of maternal psychosocial stress^17^. Our results showed no relation between maternal psychosocial stress and the microbial groups previously found to differ in infants, i.e. Proteobacteria, Actinobacteria and lactobacilli^17^, which seems to disprove our assumption. However, given that we did not investigate the infants’ intestinal microbiota, we cannot discard the hypothesis that maternal psychosocial stress affects the child via intestinal microbiota. Furthermore, it should be noted that we did find associations between general anxiety and maternal microbiota at the genus level.

Results showed that the fecal microbiota of mothers with lower prenatal anxiety was characterized by higher relative abundance of the genus *Oscillospira* and other genera from the phylum of Firmicutes (*Eubacterium* and *Megamonas*). In contrast, the fecal microbiota of mothers with higher prenatal anxiety was characterized by higher relative abundance of *Oxalobacter* (belonging to the phylum of *Proteobacteria*), *Rothia* (belonging to the phylum of *Actinobacteria*) and genera from the phylum of Firmicutes, including *Acetitomaculum*, *Acidaminococcus*, *Staphylococcus*, and unidentified genus-level taxa within the families *Peptococcaceae* and *Peptostreptococcaceae*. Of these taxa, particularly the two unidentified genera within the families *Peptostreptococcaceae* and *Peptococcaceae* were detected in the samples of almost every mother, whereas others were only found in a few samples, explaining their low average relative abundance.

*Peptostreptococcaceae* are a family of bacteria from the class Clostridia^22^. They appear to be over-represented in colorectal cancer patients^23^. *Peptostreptococcaceae* were also related to poor cognition and neuro-inflammation in cirrhosis patients with brain dysfunction^24^. Animal research shows that *Peptostreptococcaceae* might also be related to stress^25^. However, these studies suggested that high levels of stress are associated with a decrease in the relative abundance of *Peptostreptococcaceae*. For example, after treatment with antibiotics, the relative abundance of *Peptostreptococcaceae* significantly decreased in chronically stressed rats, compared to control animals^25^. Furthermore, members of the *Peptostreptococcaceae* seem to be strongly affected by diet^26,27^. For example, in adult pigs, the relative abundance of *Peptostreptococcaceae* increased when dietary protein intake decreased from 16% to 13%^26^. Also, mice fed with either a low-fat diet or high-fat diet showed reduced *Peptostreptococcaceae* when calories were restricted^27^. *Peptostreptococcaceae* were also found negatively related to life-span, regardless of fat intake^27^. Since we did not measure dietary habits, we do not know whether the difference in relative abundance in *Peptostreptococcaceae* between the low and high anxiety group is based on differences in food intake, or indeed due to differences in anxiety levels.

*Peptococcaceae* are also from the class Clostridia. *Peptococcaceae* have complex nutritional requirements, they may or may not ferment carbohydrates, and they are found in normal and pathologic female urogenital tracts^28^. Hence, vaginally delivered infants may be exposed to them during birth. *Peptococcaceae* may or may not be pathogenic^29^. Changes in urogenital bacteria in mothers might therefore expose a neonate’s gut to potentially pathogenic bacteria. However, human studies on *Peptococcaceae* are rare. Animal studies revealed a possible relationship of *Peptococcaceae* with stress and diet^30,31^. In rats reared under severe crowding stress, *Peptococcaceae* were increased compared to control rats^30^. Furthermore, high fat diet and induced stress were related to increases in *Peptococcaceae* in female rats^31^.

As said previously, we also detected differences between mothers with low and high general anxiety in genus level bacteria besides *Peptococcaceae* and *Peptostreptococcaceae*. *Megamonas*, *Eubacterium*, and *Oscillospira* were detected in either one or two samples from mothers with low general anxiety, and in none of the samples from mothers with high general anxiety. *Rothia* and *Oxalobacter* were each detected in one sample from mothers with high general anxiety, and in none of the samples from mothers with low general anxiety. *Staphylococcus* and *Acetitomaculum* were detected in, respectively, one and two samples from mothers with high general anxiety, and each in one of the samples from mothers with low general anxiety. As these bacteria were detected in only a small number of samples, we will refrain from (over-) interpreting these results. Confirmation of the findings in larger study populations is needed before an in-depth discussion of the results is warranted. Additionally, it would be important to investigate whether these bacteria are also related to anxiety in a non-pregnant population.

Our results did not provide evidence of an association between general stress or pregnancy-specific stress and anxiety and microbial abundances. It is difficult to explain why in our sample maternal gut bacteria were specifically associated to self-reports of general anxiety and not to self-reports of general stress or pregnancy-specific stress and anxiety. A possible explanation may lie in the nature of the questionnaires used. The anxiety questionnaire used in our study requires reporting on current feelings, while the other questionnaires ask about feelings over a given period of time. A questionnaire on momentary emotions may be more reliable and closely linked to reality than questionnaires that require the participant to ‘summarize’ emotions over a longer period of time and that are automatically subject to problems of recall^32^. Another explanation could be that the questionnaires about general stress and pregnancy-specific stress and anxiety asked about rather specific events, while the questionnaire on general anxiety required reporting on feelings (e.g. feelings of anxiety, nervousness). It might be that the questionnaires about stress and pregnancy events were too specific, resulting in women scoring low in the absence of such specific events, even though they may have been feeling stressed. In addition, the subscale ‘Fear of giving birth’ of the Pregnancy specific Anxieties Questionnaire-Revised had low scale reliability. Though this subscale only contains three items, and scale reliability is generally low with only a few items^33^, the subscale has been found valid in previous research^34,35^. Future research could use different questionnaires to assess general stress and pregnancy-specific stress and anxiety, to further investigate whether these types of stress are indeed not associated with microbial abundances during pregnancy.

As we only found an association between general anxiety and maternal intestinal microbiota, whereas we did not find an association between psychosocial stress and intestinal microbiota previously found to be different in infants from mothers with psychosocial stress, it is essential to discuss other potential links between prenatal stress and infant microbiota. One of these links might be related to cortisol. Cortisol concentrations in plasma increase when humans are confronted with stress and the HPA-axis is activated^5^. Maternal cortisol is known to cross the placenta and to increase cortisol concentrations in the fetus^36^. In turn, these heightened cortisol levels can affect the developing HPA axis of the fetus, resulting in increased basal cortisol concentrations and cortisol reactivity in the infant after birth^37^. Cortisol, in turn, can change the permeability of the gut and affect the immune cells in the gut, affect gut motility and secretion, and produce increases in bile acid, all of which can potentially influence the infant intestinal microbiota^17,38,39^. As cortisol may affect the maternal microbiota, and cortisol would rise as a result of maternal stress, we recommend that future studies include measures of maternal cortisol (e.g., cortisol reactivity, diurnal cortisol or chronic cortisol concentrations measured in hair). These measures would help uncover potential unique and combined (mediation) effects of psychosocial stress and cortisol on maternal microbiota during pregnancy.

Another physiological route for maternal prenatal psychosocial stress to affect offspring microbiota might take place in the postpartum period. If maternal prenatal stress continues after birth as high postnatal psychosocial stress, it might affect breast milk composition, including breast milk cortisol concentrations^40^. Maternal plasma cortisol is transferred to maternal breast milk^41^. This cortisol from milk arrives in the infants’ intestines^40^, where it binds to cortisol receptors, influencing the maturation of the gastrointestinal tract^40^, which may in turn affect which bacterial species establish themselves in the gut.

As far as we know, this is the first study to look at psychosocial stress and microbiota composition during the third trimester of pregnancy. A positive feature of the study is that several distinct aspects of psychosocial stress (i.e. general stress and anxiety, and pregnancy-specific stress and anxiety) were distinguished. Nonetheless, the study also has limitations. First, the sample consisted of mothers from a highly educated background. The current findings might not be generalizable to the whole population. Second, information on food intake was not included. As for example *Peptostreptococcaceae* and *Peptococcaceae*- which were more abundant in the high anxiety group- have previously been associated with diet, including maternal diet information in future studies will help obtain a more comprehensive understanding of observed differences in microbial community composition. In a sufficiently large sample, complex models of associations between maternal psychosocial stress and diet during pregnancy, and intestinal microbiota can be investigated. Relatedly, information on variables such as maternal smoking, history of stressful life events and social support was not collected. These variables could potentially be associated with both maternal anxiety and maternal intestinal microbiota^5^. Therefore, future studies should assess this information as well. Third, the relatively small sample size of this study might have lowered statistical power, with a risk of increasing the likelihood of Type I and Type II errors (i.e., false positive and false negative findings). Finally, as we obtained one fecal sample, changes over time and comparisons across and beyond pregnancy were not possible.

The current study offers several suggestions for future directions. First, to begin to uncover indicators of causal relationships, it would be interesting to investigate whether the associations between general anxiety and microbial composition are specific to pregnancy, whether and how the microbiota changes throughout pregnancy, and whether these changes are related to maternal psychosocial stress. Relatedly, future research is needed to find out whether the current results can be replicated in a non-pregnant female sample. Second, the field is moving towards a more complete analysis of the downstream consequences of alterations in commensal microbes, and thus future studies should also include downstream consequences (e.g., immune alterations, metabolomic alterations) of altered microbiota.

This study investigated the first step of a mechanism potentially underlying links between maternal prenatal psychosocial stress and infant outcomes, namely the relation between psychosocial stress and fecal microbiota in pregnant mothers. Contrary to our hypotheses, we did not find that mothers with high psychosocial stress had phylum-level microbial compositions characterized by more Proteobacteria, and less Actinobacteria, and lower levels of the genus *Lactobacillus*, compared to mothers with low reported psychosocial stress. However, we did find a significant association between late pregnancy general anxiety and the women’s fecal microbial composition at genus level. More specifically, the fecal microbiota of mothers with lower anxiety was characterized by higher relative abundances of the genera *Eubacterium* and *Oscillospira* compared to mothers with higher prenatal anxiety. These bacteria have been previously termed beneficial microbes. Additionally, mothers with higher prenatal anxiety had higher relative abundances of unidentified genera within the families *Peptostreptococcaceae* and *Peptococcaceae*. Previous studies have associated these bacterial groups to stress and poor health in rats and mice. Finally, we also found differences between mothers with low and high anxiety in bacteria that have not been associated with anxiety in earlier studies. The current study therefore offers insights into associations between maternal mental health and gut microbial composition during pregnancy and provides a starting point for future investigations in which maternal diet as well as infant microbiota and development should also be assessed.

## Methods

### Participants

Participants were part of the *BINGO* (Dutch acronym for *Biological Influences on Baby’s Health and Development*) study, an ongoing longitudinal study investigating prenatal predictors of infant health and development. This study was approved by the ethical committee of the Faculty of Social Sciences of the Radboud University [ECSW2014-1003-189] and was conducted according to their guidelines and regulations. Participants signed up via the project’s website, or folders that were handed out in midwife practices, pregnancy courses, and baby stores in the region Arnhem-Nijmegen (the Netherlands). Participants received a voucher with a value of 20€ and two small presents for the baby. Maternal exclusion criteria were: twin pregnancy, drug use, regular alcohol consumption, and insufficient knowledge of the Dutch language. A total of 87 expectant mothers enrolled for the study and signed the informed consent form. Of these, 73 were able to collect a stool sample. Three mothers took antibiotics at the time of collection and were therefore excluded. Subsequently, 70 healthy mothers participated in the part of the project reported here.

### Procedure

After expectant mothers signed up for the project, they completed a demographics questionnaire and a questionnaire on general anxiety. The expectant mothers were then invited for a laboratory session, which took place during the third trimester of pregnancy (*M*_*pregnant*_ = 33.9 weeks, *SD*_*pregnant*_ = 2.3 weeks). During the laboratory visit, they completed additional self-report questionnaires, including the remaining questionnaires on prenatal psychosocial stress, and performed two computer tasks and an interaction task not relevant for the current study.

Prior to the lab visit, expectant mothers collected a stool sample using a sterile stool vial (80 × 16.5 mm) with a spoon attached to the lid (Sarstedt inc.). The mothers were asked to fill one-third of the vial and to immediately store the vial in their home freezers (i.e., fresh frozen collection) until collected by the researcher. After collection, samples were stored at −80 °C until analysis. Mothers were also asked to provide information on whether they were currently ill or had been ill the previous week, whether they had used antibiotics in the past three months, and whether they took food supplements during pregnancy.

### Measures

#### Maternal psychosocial stress

In this study, to measure prenatal psychosocial stress, expectant mothers were asked to fill in questionnaires related to general, as well as pregnancy-related stress and anxiety.

#### General stress

General stress was measured with the Alledaagse Problemen Lijst (Everyday Problem Checklist; EPL^42^), a Dutch questionnaire that assesses the occurrence and intensity of daily hassles. This questionnaire contains 49 events, and participants have to check whether each event had occurred in the past two months, and if so, how much the event had bothered them on a 4-point Likert scale (1 = *not at all*, 4 = *a lot*). Subsequently, the mean intensity rating of daily hassles was calculated as the sum of how much the events bothered the participant divided by the frequency of the events. Hence, this variable could range from 0 to 4, with higher values indicating more experienced negativity as a result of daily hassles. Scale reliability (i.e., how closely related the set of items of the questionnaire are related as a group) in this sample was good, with Cronbach’s α, a measure of internal consistency, equal to 0.88.

#### General anxiety

To measure general anxiety, the state items from the State Trait Anxiety Inventory (STAI^43^) were used. The STAI is the most widely researched and used questionnaire to measure general anxiety that has proven high internal consistency^44^. Furthermore, it is relatively brief and easy to answer. The STAI questionnaire consists of 20 statements related to feelings of anxiety, yielding a score of how the participant feels at the present moment. Answers are given on a four-point Likert scale, ranging from 1 = *not at all* to 4 = *a lot*. Answers were summed up, hence scores could range from 0 to 80, with higher scores reflecting more general feelings of anxiety. Reliability of this scale in the current sample was good, with Cronbach’s α = 0.87.

#### Pregnancy-related stress

Pregnancy related stress was measured with the Pregnancy Experience Scale (PES^45^). This scale contains 43 pregnancy specific experiences. Participants are asked to rate the degree to which each experience constitutes both a hassle and an uplift during the whole pregnancy, both rated on a 4-point scale (0 = *not at all*, 3 = *totally*). Scores were derived by calculating the ratio of hassles to uplifts, i.e., the sum of intensities of hassles divided by the sum of intensities of uplifts. Scores could thus range from 0 to 3, and higher scores indicate greater negative emotional valence towards pregnancy. Scale reliability in this sample was good, Cronbach’s α = 0.89.

#### Pregnancy-related anxiety

Anxiety related to pregnancy was measured with two subscales of the Pregnancy specific Anxieties Questionnaire-Revised (PRAQ-R^34^). These subscales measure ‘fear of giving birth’ (3 items), and ‘fear of bearing a handicapped child’ (4 items) during the whole pregnancy. Items could be answered on a scale from 1 = *not at all true* to 5 = *totally true*. For ‘fear of giving birth’ scores could range between 0 and 15; for ‘fear of bearing a handicapped child’ scores could range from 0 to 20. Higher scores indicate higher levels of pregnancy-related anxiety. Cronbach’s α scale reliability was 0.52 for fear of giving birth, and 0.85 for fear of bearing a handicapped child.

#### Maternal microbiota

Approximately 0.1–0.15 g of fecal sample from each participant was used for DNA extraction. Total microbial DNA was extracted using the Maxwell® 16 Total RNA system (Promega) with Stool Transport and Recovery Buffer (STAR; Roche Diagnostics Corporation, Indianapolis, IN). Briefly, the fecal sample was homogenized with 0.25 g of sterilized 0.1 mm zirconia beads and three glass beads (2.5 mm) in 350 µL STAR buffer for 3 × 1 min at 5.5 ms using a Precellys 24 beadbeater (Bertin technologies, France). Samples were then incubated with shaking at 100 rpm for 15 min at 95 °C and pelleted by 5 min centrifugation at 4 °C and 14000 g. The supernatant was removed and the pellets were processed again as described above using 200 µL of fresh STAR buffer. The supernatant was removed, pooled with the first supernatant, and 250 µL were used for purification with Maxwell® 16 Tissue LEV Total RNA Purification Kit customized for fecal DNA extraction (AS1220) following the manufacturer’s instructions. DNA was eluted with 50 µL of DNAse and RNAse free water (Qiagen, Hilden, Germany). DNA concentrations were measured spectrophotometrically with a NanoDrop ND-1000 (NanoDrop® Technologies, Wilmington, DE, USA) and adjusted to 20 ng/µL with DNAse and RNAse free water. The V4 region of 16 S ribosomal RNA (rRNA) gene was amplified as described before^46^. PCR reactions were done in duplicate, each in a total volume of 50 µL and containing 20 ng of template DNA. Each sample was amplified with a unique barcoded primer 515F-n and 806R-n (10 µM each/reaction^40^), 1× HF buffer (Finnzymes, Vantaa, Finland), 1 µL dNTP Mix (10 mM each, Roche Diagnostics GmbH, Mannheim, Germany), 1 U Phusion® Hot Start II High Fidelity DNA Polymerase (Finnzymes, Vantaa, Finland) and 36.5 µL of DNAse and RNAse free water. The amplification program included a 30 s initial denaturation step at 98 °C, followed by 25 cycles of denaturation at 98 °C for 10 s, annealing at 56 °C for 10 s and elongation at 72 °C for 10 s, and a final extension at 72 °C for 7 min.

The PCR product presence and size (~290 bp) was confirmed with gel electrophoresis using the Lonza FlashGel® System (Lonza, Cologne, Germany). Seventy unique barcode tags were used in each library, and artificial control (Mock) communities were included. PCR products were purified with the HighPrep® PCR kit (MagBio Genomics, Alphen aan den Rijn, Netherlands), and DNA concentrations were measured with the Qubit® dsDNA BR Assay Kit (Life Technologies, Leusden, Netherlands). From each barcoded sample, 100 ng was added to the amplicon pool that was then concentrated with the HighPrep® PCR kit to 20 µL. The concentration was measured with the Qubit® dsDNA BR Assay Kit and adjusted to 100 ng/µL final concentration. The pooled libraries were sent for adapter ligation and Illumina HiSeq sequencing at GATC-Biotech, Konstanz, Germany.

Data processing and analysis were carried out using the NG-Tax pipeline^46^. Alpha diversity analyses were carried out in QIIME with rarefication cutoff of 3000 reads^47^. Principal components analysis (PCA) was performed in CANOCO 5^48^.

### Statistical analyses

Partial least squares (PLS) regression^49^ was used to assess to what extent the maternal stress variables (‘response’) could be predicted based on microbial community composition data. Briefly, PLS regression is a generalization of multiple regression, which searches for a set of components that performs a simultaneous decomposition of the predictor matrix (X = microbiota) and of the response matrix (Y = stress index), with the constraint that these components explain as much as possible of the covariance between X and Y. The optimal number of PLS components was defined using double-cross validation^50^. One component was found to be appropriate to model the data, and the R^2^ parameter (variance explained, where R^2^ = 1 indicates perfect prediction ability of the model) was used to assess the quality of the final regression model. Model significance was assessed using a permutation test with 1000 permutations, with significance cut-offs of 0.01 and 0.05. We corrected for multiple testing with Bonferroni. All p-values presented are after Bonferroni correction. Selection of the most important bacteria (i.e., bacteria that were detected in most samples, and drive the results) in the final model was performed using the Significance Multivariate Correlation criterion^51^. Data (X) were centered and scaled to unit variance; the response Y was log transformed before analysis. PLS was performed using the Matlab MEDA-toolbox^52^.

Shannon and Chao1 scores from alpha diversity analyses were calculated in QIIME and two-tailed, unpaired t-tests were then used to compare the scores between mothers in the high and low groups (i.e. above and below the median, respectively) for the maternal stress variables that were significantly associated with bacterial relative abundance profiles in mothers.

Finally, we performed Principal Coordinate Analysis (PCoA) based on weighted or unweighted unifrac distances in QIIME, and Principal Component Analysis (PCA) and Redundancy analysis (RDA) based on relative abundance distributions using Canoco 5 software to check for sample clustering at genus level in relation to the maternal stress variables that significantly predicted bacterial clustering.

## Data Availability

The datasets generated during and/or analyzed during the current study are not publicly available due to them being part of an ongoing longitudinal study, but are available from the corresponding author on reasonable request.
